# Estimation of myocardial infarction death in Iran: artificial neural network

**DOI:** 10.1186/s12872-022-02871-8

**Published:** 2022-10-07

**Authors:** Mohammad Asghari-Jafarabadi, Kamal Gholipour, Rahim Khodayari-Zarnaq, Mehrdad Azmin, Gisoo Alizadeh

**Affiliations:** 1 Cabrini Research, Cabrini Health, Melbourne, VIC 3144 Australia; 2 School of Public Health and Preventative Medicine, Faculty of Medicine, Nursing and Health Sciences, Monash University, Clayton, VIC 3800 Australia; 3 Road Traffic Injury Research Center, Tabriz University of Medical Sciences, Tabriz, Iran; 4 Tabriz Health Service Management Research Center, School of Management and Medical Informatics, Tabriz University of Medical Sciences, Tabriz, Iran; 5 Department of Health Policy and Management, School of Management and Medical Informatics, Tabriz University of Medical Sciences, Tabriz, Iran; 6 Non-Communicable Diseases Research Center Endocrinology and Metabolism Population Sciences Institute, Tehran University of Medical Sciences, Tehran, Iran

**Keywords:** Estimation, Death rate, Myocardial infarction, Artificial neural network, Iran

## Abstract

**Background:**

Examining past trends and predicting the future helps policymakers to design effective interventions to deal with myocardial infarction (MI) with a clear understanding of the current and future situation. The aim of this study was to estimate the death rate due to MI in Iran by artificial neural network (ANN).

**Methods:**

In this ecological study, the prevalence of diabetes, hypercholesterolemia over 200, hypertension, overweight and obesity were estimated for the years 2017–2025. ANN and Linear regression model were used. Also, Specialists were also asked to predict the death rate due to MI by considering the conditions of 3 conditions (optimistic, pessimistic, and probable), and the predicted process was compared with the modeling process.

**Results:**

Death rate due to MI in Iran is expected to decrease on average, while there will be a significant decrease in the prevalence of hypercholesterolemia 1.031 (− 24.81, 26.88). Also, the trend of diabetes 10.48 (111.45, − 132.42), blood pressure − 110.48 (− 174.04, − 46.91) and obesity and overweight − 35.84 (− 18.66, − 5.02) are slowly increasing. MI death rate in Iran is higher in men but is decreasing on average. Experts' forecasts are different and have predicted a completely upward trend.

**Conclusion:**

The trend predicted by the modeling shows that the death rate due to MI will decrease in the future with a low slope. Improving the infrastructure for providing preventive services to reduce the risk factors for cardiovascular disease in the community is one of the priority measures in the current situation.

## Introduction

Cardiovascular disease is the leading cause of morbidity, mortality, and disability worldwide. Despite the significant advances that have been made today in the field of prevention and control of cardiovascular diseases, these diseases are still one of the leading causes of death in the world [1–3]. At the beginning of the twentieth century, cardiovascular disease was responsible for 10% of all deaths in the world, but in the twenty-first century, it is responsible for 50% of deaths in developed countries and 25% of deaths in developing countries [4]. In 2012, two-thirds of deaths were due to non-communicable diseases related to cardiovascular disease and cancer [5]. The number of deaths due to heart disease is projected to increase from 17.5 million in 2012 to 22.2 million in 2030 if the current trend continues [6]. The world is projected to lose about $47 trillion due to non-communicable diseases between 2011 and 2030, of which about $30 trillion will be due to four diseases: diabetes, cancer, chronic lung disease, and cardiovascular disease [7]. Cardiovascular disease, including heart attacks and strokes, accounts for one-third of all deaths in the United States, with $315 billion spent annually on health care and lost productivity costs [8]. The prevalence of non-communicable diseases is also increasing in Iran [9]. It is also predicted that the burden of cardiovascular patients will increase by 2025 due to the aging population in Iran [10].

Risk factors of non-communicable diseases include behavioral, psychological, and biological. Findings of various studies show that psychological and behavioral risk factors, along with biological risk factors, have an effect on the incidence and progression of MI, which should be given special attention in prevention programs [11, 12]. In recent years, the risk factors for non-communicable diseases such as smoking, obesity, high blood pressure, inadequate physical activity, and consumption of inappropriate foods, all rooted in poor lifestyle, have also increased in Iran [13–15]. As predicted in Iran according to the report of the World Health Organization; The death rate from cardiovascular disease will reach 44.8% by 2030 [2, 10, 16, 17].

Despite significant advances in the diagnosis and treatment of this disease, MI remains the most common cause of heart failure (HF) [18]. In fact, cardiovascular disease is an epidemic of our time that will cause many disabilities and treatment costs. It is essential to have thorough knowledge of the illness's status and be aware of the factors influencing the disease burden in order to decide on the optimal intervention program and decision to lower the burden of CVD [19]. Predictive studies have provided access to such information. Recent years have seen an increase in the adoption of future research, including modeling, particularly in the field of health policy. Modeling studies have been widely utilized to assess the potential significance of variables with incomplete or no data [20]. Such approaches (future studies) are crucial for resource allocation, management, and priority in low- and middle-income nations since they have little resources in terms of money, time, and human capital [21]. The achievement of national and international objectives can be aided by forecasting the future of myocardial infarction in Iran. According to modeling and expert opinion and health policy makers can use the expected trends in their planning and policy-making. The aim of this study was to estimate the death rate due to MI in Iran by Artificial Neural Network.

## Methods

This study is ecological and data on the prevalence of diabetes, hypercholesterolemia over 200 (mg/dL^1^), hypertension, overweight and obesity for the years 1990–2017 were extracted from the STEPs study and information on stroke death (per 10,000 population) for the years 1990–2016 [22], Extracted from NASBOD study [23–25]. These risk factors will be selected according to a model proposed by American Heart Association [26].

ANN have been given attention in predicting the effects of multiple variables with complex relationships on a specific variable. ANN have attracted a lot of attention in the last few years [27]. Artificial neural networks are algorithms that can be used to perform nonlinear statistical modeling and provide a new alternative to logistic regression, the most commonly used method for developing predictive models for dichotomous outcomes in medicine. Neural networks offer a number of advantages, including requiring less formal statistical training, ability to implicitly detect complex nonlinear relationships between dependent and independent variables, ability to detect all possible interactions between predictor variables, and the availability of multiple training algorithms [28].

First, the normality of the data was assessed using the Kolmogorov–Smirnov test. Then, using the artificial neural network method, the prevalence of diabetes, cholesterol over 200, hypertension, overweight and obesity for the years 2017–2025 were estimated. Artificial neural networking technique was also used to predict the future. Independent variables for Input (prevalence of diabetes, hypercholesterolemia over 200, hypertension, overweight and obesity) and dependent variable (death rate per 100,000 population) were considered as Output and also a 5% error was considered. Also, in the present study, there is no distortion variable in the study. Linear regression model was used to determine the factors related to myocardial risk factors. In statistical analysis, p < 0.05 was considered significant. Data was analyzed using the Statistical Package for the Social Sciences (SPSS) software for Windows, (version 17) (SPSS Inc., Chicago, IL, USA).

cThen, by providing information about the risk factors and deaths due to myocardial infarction in recent years in the form of a questionnaire, experts were asked to consider the condition of death in 3 cases (optimistic, pessimistic and probable). 13 experts with the following specifications completed the submitted questionnaire (Table 1).

**Table 1:** Profile of participants in predicting the death rate due to myocardial infarction

No	Gender		Degree of education	Field of Study	Job
	Male	Female			
1	*		Ph.D	Epidemiology	Science committee
2	*		Ph.D	Health economics	Science committee
3	*		Ph.D	Health management services	Science committee
4		*	Ph.D. student	Health policy making	Student
5	*		Ph.D	Health policy making	Science committee
6		*	Ph.D	Health promotion	Science committee
7	*		Ph.D. student	Epidemiology	Student
8	*		Ph.D	Elderly science	Science committee
9	*		Ph.D	Health services management	Science committee
10	*		Ph.D	Elderly science	Science committee
11	*		Ph.D. student	Health management services	Student
12		*	Ph.D	Health policy making	Science committee
13	*		Ph.D. student	Health policy making	Student

Predict a heart attack per 100,000 people. The predicted trend based on expert opinion was compared with the modeling trend based on ANN.

## Results

The prevalence of diabetes, hypercholesterolemia, hypertension, overweight and obesity per cent and deaths from stroke (per 10,000 population) were estimated for the years 2017–2025. Findings show that the prevalence of diabetes is upward in all age groups and in men and women. In men and women, the prevalence of diabetes in the age groups 65–69, 70–74, 75–79, 80–84 and 85 years and above will increase with a steeper slope. Figures are drawn with the help of Graph Pad Prism 8.4.3 software.

**Fig. 1:**
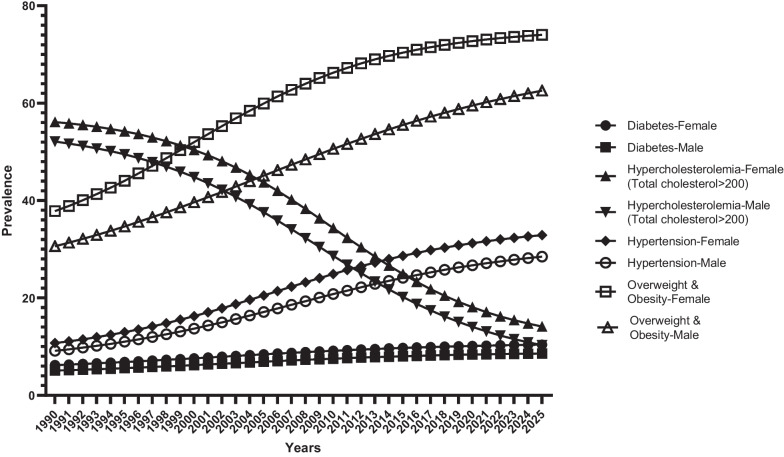
Prevalence of risk factors, Age-Standard (percentage)

The prevalence of hypercholesterolemia > 200 in women and men is declining, unlike other risk factors (diabetes, hypertension, obesity, and overweight). A review of data from the last 25 years (1990–2015) also shows a downward trend. From 2016 to 2025, a downward trend with a low slope is also predicted based on Fig. 1.

**Fig. 2:**
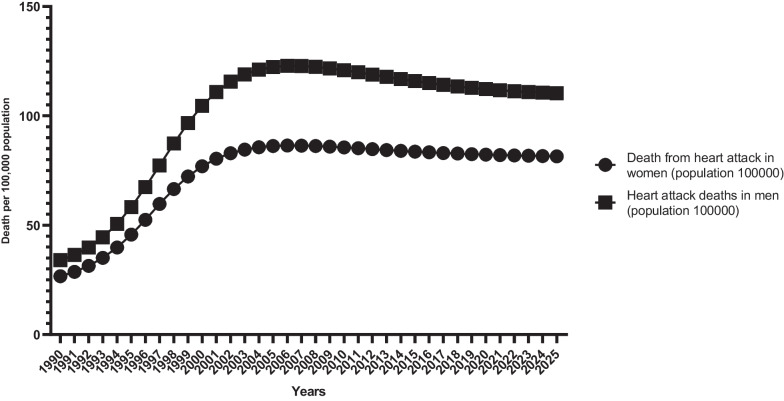
MI deaths—Age-Standard (per 100,000)

The prevalence of hypertension in men and women such as diabetes, obesity and overweight are on the rise. A review of data from the last 25 years (1990–2015) also shows an upward trend. Prevalence is highest in men and women 85 years of age and older. The prevalence of obesity and overweight varies in age groups. The prevalence is highest in men and women in the age groups of 50–54, 55–59, 65–69 and 60–64. Examination of the death rate due to stroke shows that until 2005 it was an upward trend with a peak and then a downward trend. Examination of the death rate due to MI shows that until 2005 it was an upward trend with a peak and then a downward trend. For 2016–2025, a downward trend with a low slope is also predicted (Fig. 2).

Then, by providing information about the risk factors and deaths due to myocardial infarction in previous years, experts were asked to predict the death rate due to myocardial infarction per 100,000 population, taking into account the conditions of the three selected scenarios. The predicted trend by experts was compared with the modeling trend. 13 specialists with the following specifications completed the submitted questionnaire. 77% of the participants were male. Participants' majors included epidemiology, health economics, health care management, health policy, health education and health promotion, and geriatrics.

**Fig. 3:**
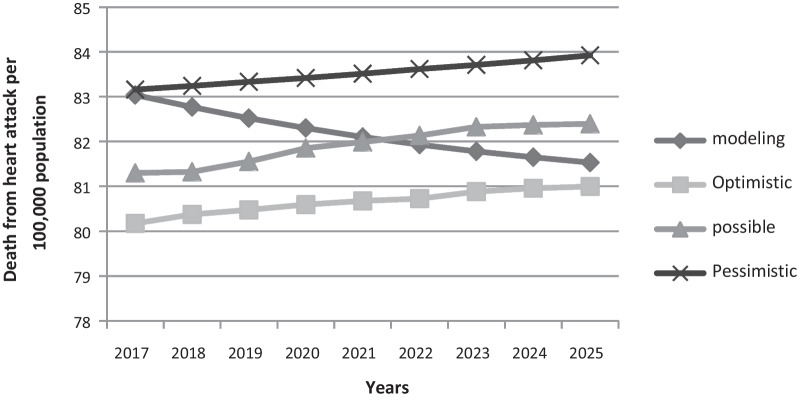
Comparison of predicted deaths in different scenarios due to myocardial infarction in 100,000 populations in women—Age-Standard

**Fig. 4:**
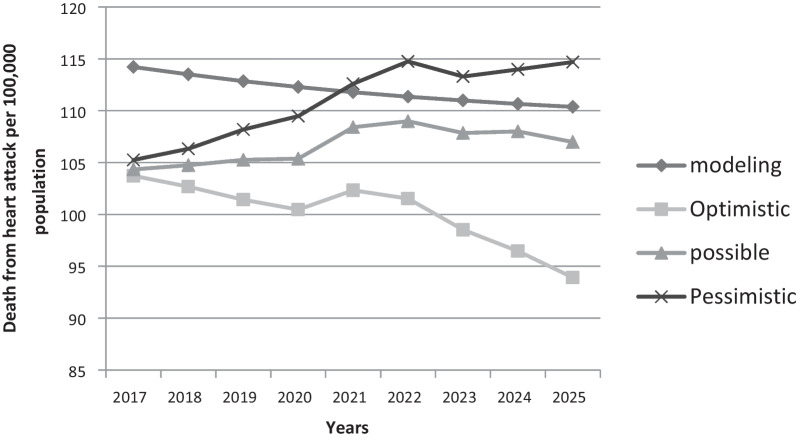
Comparison of death rates due to myocardial infarction in 100,000 population in men- Age-Standard

Comparison of deaths due to MI per 100,000 population in age-adjusted men and women shows that experts in the optimistic scenario lower the death rate than the modeled trend, in the pessimistic scenario the uptrend and above the modeled trend, and in the scenario Probably predicted the trend close to the modeled trend (Figs. 3, 4).

Result of regression of death due to myocardial infarction per 100,000 populations is associated with blood pressure, hypercholesterolemia > 200 and overweight and obesity in men and women in the years 1990–2016. In the years 2017–2025, age, hypertension and overweight and obesity in men and age and blood pressure in women are associated with death due to MI per 100,000 populations. It is expected that in the period from 2017 to 2025, the death rate per 100,000 will be 771 0.09 units higher among males of each age group than in the younger age group; among females, this number is equal to 1037.65 units (P < 0.001). Prevalence of hypertension and overweight and obesity among males and prevalence of hypertension among females were statistically significant predictors of death (Table 2).

**Table 2:** Results of multiple Regression model of the predictors of MI Death rate

Variable	Male				Female			
	Coefficient	Coefficient 95% confidence interval		*P* value	Coefficient	Coefficient 95% confidence interval		*P* value
		Upper limit	Low limit			Upper limit	Low limit	
*1990–2016*								
Age	6.57	61.32	− 48.17	0.81	34.70	− 42.72	112.1	0.37
Diabetes	34.31	− 39.17	20.86	0.193	53.19	7.32	99.06	0.023
blood pressure	46.91	30.13	63.69	< 0.001	23.55	9.15	37.95	0.001
Hypercholesterolemia > 200	− 43.89	− 57.68	− 30.10	< 0.001	− 75.26	− 83.83	− 66.68	< 0.001
Overweight and obesity	− 39.39	− 49.15	− 29.63	< 0.001	− 25.38	− 35.77	− 15	< 0.001
*2017–2025*								
Age*	771.09	604.72	937.46	< 0.001	1037.65	869.82	1205.48	< 0.001
Prevalence of diabetes	10.48	− 11.45	13.42	0.86	16.63	− 65.73	99	0.69
Prevalence of hypertension	− 110.48	− 174.04	− 46.91	0.001	− 129.03	− 168.14	− 89.92	< 0.001
Prevalence of hypercholesterolemia > 200	1.31	− 24.81	26.88	0.937	17.79	− 1.82	37.42	0.075
Prevalence of overweight and obesity	− 35.84	− 52.02	− 18.66	< 0.001	− 13.21	− 32.56	6.12	0.178

Dependent variable: death rate in 100,000

*Age is classified into 5-year groups (1 = 25–29 years old, 13 ≥ 85 years old)

## Discussion

According to the modeling results, the mortality rate due to myocardial infarction in Iran is expected to decrease on average, while there will be a significant reduction in the prevalence of hypercholesterolemia > 200. Also, the trend of diabetes, blood pressure and obesity and overweight will increase slowly and this trend will continue until 2025. The death rate from myocardial infarction in Iran is higher in men but is decreasing on average.

The global prevalence of diabetes among adults (20 to 79 years) in the world in 2010 (285 million) is 6.4% and is projected to increase to (439 million) by 7.7% by 2030 [29]. And this trend is predicted in another study to increase to 693 million by 2045 [30]. The results of modeling show that the trend of diabetes prevalence in Iran by 2025 is upward and is consistent with global studies.

The results of the present study show that the prevalence of hypercholesterolemia in both sexes (male and female) will decrease by 2025. A study conducted in China to study the achievement of sustainable development goals by 2030 shows a declining trend in hypercholesterolemia [31]. But no study was found to examine the future trend of hypercholesterolemia.

High blood pressure is a preventable risk factor for cardiovascular disease, which has a high prevalence in society, and modeling results show that the prevalence of high blood pressure will increase by 2025. The results of a study in India show that the prevalence of hypertension has increased among the urban population of India over the past 25 years, and the forecast shows that this trend will continue until 2030 [32]. A study by Pandi et al. In Nepal predicted the prevalence of hypertension using the simulation method by 2040. The results showed that the prevalence of hypertension in this country will also be upward [33]. Another study, which examined the prevalence of hypertension in urban areas by 2030 with an analysis of 32 studies, predicted that the prevalence of hypertension would be upward and to 32% in women, 36.5% in men and 34.5% in general [34]. The results of other studies confirm the modeling results and the upward trend in the prevalence of hypertension in Iran.

Obesity has a great impact on the risk factors for cardiovascular disease. A body mass index above 22.5 can lead to cardiovascular disease and high blood pressure. A study by Pandi et al. In Nepal predicted the prevalence of obesity using the simulation method, which shows the prevalence of obesity up to 2040 with a steep slope [33]. Findings from a study in the United States show that by 2030, approximately one in two adults will be obese, with a prevalence of more than 50 percent in 29 states and no less than 35 percent in any state [35]. A study of the trend of obesity in Iran between 2007 and 2011 shows that the prevalence of obesity in Iran during these years has been increasing [36]. The modeling results predict an upward trend in the prevalence of obesity for all age groups and in both gender groups by 2025.

The results of a study estimating the rate of premature death from cardiovascular disease in 188 countries between 2013 and 2025 show that the overall trend in the rate of premature death from cardiovascular disease is declining. This study indicates that by reducing the prevalence of risk factors for cardiovascular disease, it will be possible to achieve a 25% reduction in mortality by 2025 [37]. Another study in the United States found that deaths from cancer and cardiovascular disease were declining. The study also showed that the total number of deaths among white men and women decreased from 2000 to 2014, from 175,000 to 135,000 [38]. In another study to look at the mortality rate in Brazil from 2001 to 2011, the results showed that despite an increase in the total number of deaths due to cardiovascular disease, the age-adjusted mortality rate for these diseases decreased by 24% [39]. Overall, various studies of myocardial infarction in the United States, Brazil, Japan, the United Kingdom, Sweden, Canada, Ireland, and Denmark show that mortality rates have declined over the past two decades [40–44].

The results of a study that predicts deaths due to non-communicable diseases in Iran by 2030 show that the mortality rate due to cardiovascular disease is declining. It also refers to better management of risk factors, early diagnosis of the disease due to more comprehensive care in all sections of society and improving literacy and awareness throughout the country [45]. Despite the declining mortality rate from cardiovascular disease in the coming years, we should not be indifferent to prevention and control programs because the burden and direct and indirect costs of cardiovascular disease are increasing due to the aging population [10, 46].

One of the limitations of this study is that only some risk factors have been investigated. One of the reasons for the discrepancy between the trend in mortality based on data from previous years and the opinion of experts is that experts have expressed their opinion about the future trend, taking into account the circumstances of each situation. In fact, it is simplistic to hope for a reduction in the incidence and mortality of cardiovascular disease, especially heart attacks, regardless of drivers such as political stability, favorable economic and social conditions, and access to health services. On the other hand, certainly with more attention to the issue of non-communicable diseases, especially cardiovascular disease, we can see a decreasing trend of risk factors and death rate due to cardiovascular disease. The increasing progress of medical sciences in the methods of diagnosis and treatment of cardiovascular diseases, especially myocardial infarction, is significant. It is clear that this attention will reduce the mortality rate due to myocardial infarction in the coming years, and from this, the results of the study are partially justified.

## Data Availability

The datasets used and/or analyzed during the current study are available from the corresponding author on reasonable request.

## Footnotes

1 Milligrams per deciliter.
